# Galectin-3–null mice display defective neutrophil clearance during acute inflammation

**DOI:** 10.1189/jlb.3A0116-026RR

**Published:** 2016-10-12

**Authors:** Rachael D Wright, Patricia R. Souza, Magdalena B. Flak, Prasheetha Thedchanamoorthy, Lucy V. Norling, Dianne Cooper

**Affiliations:** William Harvey Research Institute, Barts and the London School of Medicine and Dentistry, London, United Kingdom

**Keywords:** lectin, efferocytosis, apoptosis, peritonitis

## Abstract

Galectin-3 has been associated with a plethora of proinflammatory functions because of its ability, among others, to promote neutrophil activation and because of the reduction in neutrophil recruitment in models of infection in Gal-3-null mice. Conversely, it has also been linked to resolution of inflammation through its actions as an opsonin and its ability to promote efferocytosis of apoptotic neutrophils. Using a self-resolving model of peritonitis, we have addressed the modulation and role of Gal-3 in acute inflammation. We have shown that Gal-3 expression is increased in neutrophils that travel to the inflamed peritoneum and that cellular localization of this lectin is modulated during the course of the inflammatory response. Furthermore, neutrophil recruitment to the inflamed peritoneum is increased in Gal-3–null mice during the course of the response, and that correlates with reduced numbers of monocytes/macrophages in the cavities of those mice, as well as reduced apoptosis and efferocytosis of Gal-3–null neutrophils. These data indicate a role for endogenous Gal-3 in neutrophil clearance during acute inflammation.

## Abstract (precis)

Expression of galectin-3 by exudated neutrophils drives neutrophil apoptosis and clearance in a model of self-resolving peritonitis.

## Introduction

Galectins are a family of β-galactoside–binding proteins that elicit their effects by binding to exposed *N*-acetyllactosamine residues on cells [1]. Several galectins have been designated with key immunomodulatory roles in a range of pathologic settings; of these, Gal-3 has been identified as a proinflammatory molecule that functions to drive the inflammatory response through the activation of innate immune cells and its chemoattractant actions [2–5]. During infections with pathogens such as *Streptococcus pneumoniae*, Gal-3 appears to be beneficial for the host, with enhanced pathogenicity observed in Gal-3–null mice and reduced infection in mice treated with recombinant Gal-3 [6, 7]. Such effects are thought to be due to Gal-3 enhancing the recruitment/activation of neutrophils as well as having direct bactericidal actions [6]. Administration of recombinant Gal-3 to human neutrophils results in degranulation, release of reactive oxygen species, and an increase in phagocytic capability [3, 5, 8]. Decreased neutrophil recruitment is seen in the lungs of Gal-3–null mice infected with *S. pneumoniae*, and that can be rescued by administration of the recombinant protein [9]; Gal-3 can also act directly as an adhesion molecule, and it promotes adhesion of neutrophils to endothelial cells in vitro [7]. The effects of Gal-3 on neutrophil trafficking appear to be specific to particular pathogens because no differences were observed in neutrophil trafficking to the lungs in Gal-3–null mice infected with *Escherichia coli* or to air pouches inoculated with the *Leishmania major* substrain Friedlin when compared with substrain LV39 [10]. Interestingly, both *S. pneumoniae* and LV39 induced release of Gal-3 into the inflammatory exudate, whereas *E. coli* and Friedlin did not, suggesting that it is the presence of Gal-3 in inflammatory exudates that functions to drive neutrophil recruitment. This hypothesis is supported by the finding that administration of recombinant Gal-3 into the murine air pouch results in neutrophil recruitment [10]. Whether endogenous Gal-3 is released and has a role in neutrophil trafficking to other sites of inflammation, such as an inflamed peritoneal cavity, is not clear. Colnot et al. [11] observed reduced recruitment of granulocytes in a model of thioglycollate-induced peritonitis in Gal-3–null mice on d 4 only, whereas Hsu et al. [12] observed reduced numbers of neutrophils on d 1 only. Furthermore, it is not clear how Gal-3 promotes neutrophil transmigration when it is released into inflammatory exudates or injected into a cavity such as the air pouch because it is not chemotactic for neutrophils [9], and it does not appear to modulate the levels of major chemokines/cytokines within inflammatory exudates [10].

As well as the aforementioned studies supporting a role for Gal-3 in driving innate immunity, there is evidence to suggest that it may facilitate resolution of inflammation through its actions as an opsonin [13]; furthermore, Gal-3–deficient macrophages are less efficient at phagocytosing apoptotic neutrophils than WT cells are [6]. Binding of Gal-3 to human neutrophils promotes the exposure of phosphatidylserine without inducing apoptosis, a process known to act as an “eat-me” signal for phagocytes, suggesting a role for Gal-3 in the clearance of neutrophils [14]. However, evidence that treatment of human neutrophils with recombinant Gal-3 delays their spontaneous apoptosis and that the rate of neutrophil apoptosis from Gal-3–null mice does not differ from that of WT cells is at odds with a proresolving function [6, 11].

Although the studies mentioned provided evidence that Gal-3 is an important regulator of the innate immune system, the modulation of the endogenous protein in innate immune cells during the course of an inflammatory response has not, to our knowledge, been studied systematically. The expression of Gal-3 is reportedly negligible basally in both human and murine neutrophils, and whether levels are modulated during inflammation is not clear because, again, contrasting reports exist. Gil et al. [15] demonstrated an increase in Gal-3 expression in recruited neutrophils after Carrageenan-induced peritonitis in the rat, whereas Sato et al. [7] showed no increase in lectin expression in murine neutrophils that had been recruited to *S. pneumoniae*–infected alveoli. Human neutrophils express Gal-3, and levels are not modulated upon migration through an endothelial monolayer in an in vitro transmigration assay in response to ILN-8 [16].

In this study, we characterized the regulation and function of Gal-3 in neutrophils during the course of a self-resolving inflammatory response. Our findings demonstrate that Gal-3 expression is increased in neutrophils that have migrated into the inflamed peritoneal cavity and that neutrophil numbers are increased in the peritoneal cavities of Gal-3–null mice. This is likely due to reduced clearance rather than increased trafficking, as evidenced by reduced levels of apoptosis in Gal-3–null neutrophils coupled with their reduced efferocytosis.

## MATERIALS AND METHODS

### Reagents

All Abs were purchased from eBioscience (San Diego, CA, USA). The Annexin V^FITC^ apoptosis detection kit was purchased from BD Biosciences (Franklin Lakes, NJ, USA). CellTrace carboxyfluorescein succinimidyl ester was purchased from Thermo Fisher Scientific (Waltham, MA, USA). Histopaque 1077 and Zymosan were purchased from Sigma-Aldrich (St. Louis, MO, USA).

### Ethics

All animal studies were conducted with ethical approval from the Queen Mary University of London Local Ethical Review Committee and in accordance with the United Kingdom Home Office regulations (Guidance on the Operation of Animals, Scientific Procedures Act, 1986).

### Mice

Male C57BL/6 mice were obtained from Charles River (Wilmington, MA, USA). Breeding pairs of Gal-3–null mice (B6.Cg-Lgals3tm1Po1/J) were provided by the Consortium for Functional Glycomics (http://functionalglycomics.org), and a colony was established in Margate, United Kingdom, at the Charles River Laboratories (Wilmington, MA, USA). These mice were on a C57BL/6 background, and age- and sex-matched controls were used for all experimental work. All animals were fed standard laboratory chow and water ad libitum and were maintained on a 12-h light–dark cycle under specific pathogen-free conditions. All experiments were performed with mice 6–7 wk old.

### Peritonitis

Briefly, mice were injected with 1 mg zymosan (Sigma-Aldrich) i.p. in 1 ml sterile PBS, as previously described by Ajuebor et al. [17] in 1999; 0-h mice received no treatment. At 4, 24, 48, 72, and 96 h after injection, the mice were anesthetized, along with the 0 h controls, with isoflurane, and a cardiac puncture was performed to obtain peripheral blood. The mice were then sacrificed by cervical dislocation, and peritoneal lavages were performed with 4 ml ice-cold PBS to collect peritoneal exudates. Tibias and femurs were collected, cleaned, and flushed with sterile PBS using a 25-guage needle to extract bone marrow. Bone marrow cells were then filtered through a 70-µm filter and washed before staining for analysis by flow cytometry.

In some experiments peritonitis was induced using 1 mg lyophilized *E. coli* (strain K12; Sigma-Aldrich) i.p. in 1 ml sterile PBS. Peritoneal cavities were lavaged, as described above, at 4 h postinjection.

### Flow cytometry

Murine cells were incubated with Abs for Ly6G (eBioscience, clone 1A8) to identify neutrophils or a combination of F4/80 and Gr-1 (eBioscience, clone BM8 and RB6-8C5, respectively) to identify classic and nonclassic monocyte/macrophages. Cells were then fixed and permeabilized with BD fixation and permeabilization buffer before the addition of the anti–Gal-3 Ab (eBioscience, clone M3/38) to assess intracellular Gal-3 levels (some cells were incubated with Ly6G and Gal-3 without permeabilization to assess cell surface levels). To determine cytosolic expression of Gal-3, mean fluorescence intensities for surface expression were deducted from the mean fluorescence intensity values obtained for total expression. In all cases, Abs or relevant isotype controls were incubated at 4°C before flow cytometric analysis using FlowJo software (Tree Star, Ashland, OR, USA).

### Confocal microscopy

Peritoneal lavage cells were seeded onto Ibidi 8-well chamber slides in RPMI 1640 and allowed to adhere. After fixation with 1% paraformaldehyde, cells were blocked with 5% FBS before permeabilization with 0.05% Triton X-100. Staining for intracellular Gal-3 was performed using anti–Gal-3–PE (eBioscience) for 1 h at room temperature. Cells were washed 3 times and then incubated with phalloidin 647 for 20 min at room temperature. Finally, cells were mounted with ProLong Gold Antifade Mountant containing DAPI (Thermo Fisher Scientific). Immunofluorescence was assessed using a Zeiss LSM 710 confocal microscope (Carl Zeiss Microscopy, Jena, Germany). Images were processed using Zen 2012 (Carl Zeiss Microscopy) and Adobe Photoshop CS6 (Adobe Systems, San Jose, CA, USA) software.

### Adoptive transfer

WT mice were euthanized, and tibias and femurs were collected, cleaned, and flushed with RPMI 1640 (10% FCS; 2 mM EDTA) using a 25-guage needle, as described previously [18]. Following centrifugation, red blood cells were lysed with 0.2% hypotonic saline for 20 s. Bone marrow cells were then washed in RPMI 1640, and the yield of neutrophils was quantified using Turk’s solution. To label cells for transfer fluorescently, neutrophils were resuspended in PBS at a concentration of 5 × 10^6^/ml; 5 µM CFSE (Thermo Fisher Scientific) was added, and cells were incubated at 37°C for 8 min. An equal volume of FCS was then added to quench excess CFSE, and the cells were washed. After 2 additional washes with ice-cold RPMI 1640, the cells were resuspended in ice-cold PBS at a density of 25 × 10^6^/ml. Then, 5 × 10^6^ neutrophils were administered to each Gal-3–null recipient mouse via the lateral tail vein, and 1 mg zymosan was injected i.p. 4 h after zymosan administration; peritoneal lavages were collected, as described above, and cells were stained with anti-Ly6G to identify neutrophils and Gal-3. Some cells were also permeabilized after the surface stain with Ly-6G to assess intracellular expression of Gal-3.

### Gal-3 ELISA

Gal-3 levels were measured in peritoneal exudates using a commercial ELISA kit (R&D Systems, Minneapolis, MN, USA) according to manufacturer’s instructions.

### Apoptosis assay

Exudated neutrophils from WT and Gal-3–null mice were collected after 4, 48, 72 and 96 h of zymosan-induced peritonitis and were labeled with Ly6G. Then, the cells were labeled with Annexin V and propidium iodide as per manufacturer’s instructions (BD Biosciences) or Zombie NIR viability dye (1:400 dilution in PBS; BioLegend, San Diego, CA, USA) to assess apoptosis. In some experiments, 4-h exudated neutrophils were cultured overnight in RPMI 1640 and assessed the following day at 18 h for surface Gal-3 levels and apoptosis. Gal-3 staining was performed as described above, and apoptosis levels were assessed using Annexin V and Zombie NIR. Viable (Annexin V^−^, Zombie^−^) and late apoptotic (Annexin V–Zombie double positive) cells were gated, and Gal-3 expression assessed.

### Efferocytosis assay

Biogel-elicited macrophages were collected from WT mice, as previously described [19], and were seeded into 24-well plates at 0.5 × 10^6^ cells/well and allowed to adhere. Exudated neutrophils from WT and Gal-3–null mice were collected after 4 h zymosan-induced peritonitis and labeled with BODIPY-FL (Thermo Fisher Scientific) for 5 min at 37°C. After washing, neutrophils were resuspended in RPMI 1640 at 2 × 10^6^/ml, and 0.5 ml was added to each well of macrophages and incubated for 1 h. After extensive washing efferocytosis was quantified using ImageJ software (U.S. National Institutes of Health, Bethesda, MD, USA). Images were split into their individual red/blue/green channels, and the same background threshold was applied to all images before quantification of fluorescence.

### Statistical analysis

Statistical significance was assessed using SPSS computer software (SPSS Inc., Chicago, IL, USA). Data are expressed as means ± sem of *n* experiments. All data were tested for normal distribution, and power calculations were performed using G*Power software (Heinrich Heine University of Düsseldorf, Düsseldorf, Germany) [20]. Statistical differences were analyzed by 1- or 2-tailed *t* test for 2 groups, 1-way ANOVA, followed by a Bonferroni or Dunnett’s post hoc test (depending on whether all values or each value, respectively, was compared with a control) or 2-way ANOVA, followed by Bonferroni post hoc test. In all cases a *P* value < 0.05 was considered significant to reject the null hypothesis, and differences were considered significant.

## RESULTS

### Modulation of Gal-3 expression in murine neutrophils during inflammation

**Figure 1.**
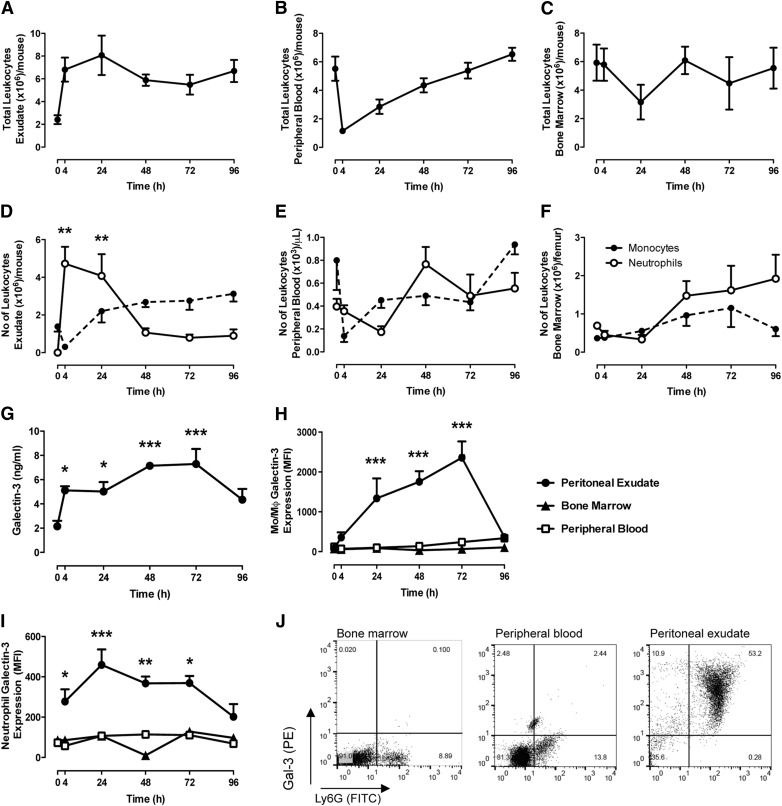
Expression of Gal-3 by murine neutrophils during a resolving inflammatory response. Total leukocyte counts in the peritoneal exudate (A), blood (B), and bone marrow (C) after zymosan (1 mg) challenge in the peritoneal cavity. Differential leukocyte counts in the peritoneal exudate (D), blood (E), and bone marrow (F) after zymosan (1 mg) challenge in the peritoneal cavity. (G) Gal-3 concentration in peritoneal exudate fluid after zymosan-induced peritonitis. Total Gal-3 expression in monocyte/macrophages (H) and neutrophils (I) after zymosan (1 mg) challenge in the peritoneal cavity. (J) Representative dot plot showing neutrophils double-stained for Ly6g and Gal-3 at 4 h after zymosan in the bone marrow, peripheral blood, and peritoneal cavity. *N* = 12/group for peritoneal exudate, 8/group for peripheral blood, and 4/group for bone marrow. MFI, mean fluorescence intensity; PE, peritoneal exudate. *P < 0.05, **P < 0.01, and ***P < 0.001 vs. peripheral blood and bone marrow at same time point or at 0-h control.

To investigate the endogenous levels of Gal-3 during the course of an acute inflammatory response, a zymosan-induced peritonitis was performed and monitored for 96 h; by which time, the inflammation had resolved. Because of the discrepancy among reports of Gal-3 expression in neutrophils, we fully investigated expression levels in 3 sources of neutrophils: those from the bone marrow, those from the peripheral blood, and those that had migrated into the inflamed peritoneal cavity. There was a rapid increase in the number of total leukocytes in the peritoneal cavity within 4–24 h after zymosan administration, and that number remained elevated during the course of the response, with increased leukocyte numbers still observed at 96 h compared with basal levels (**Fig. 1A**). The neutrophil number in the peritoneal cavity increased sharply for 4–24 h and then returned to basal levels within the 96-h time course. In contrast, the monocyte/macrophage numbers declined within the first 4 h of zymosan treatment and then repopulated the peritoneal cavity over the time course (Fig. 1D). The number of leukocytes within peripheral blood decreased rapidly after induction of peritonitis and then returned to basal levels during the course of the response (Fig. 1B). Monocytes account for the decrease in total cells seen at 4 h, which return to basal levels during the time course. Although there were no significant modulations in the numbers of neutrophils in the peripheral blood, a trend can be seen that mirrors the changes in the peritoneal cavity with a decrease as cells migrate from the peripheral blood to the peritoneal cavity and then an increase as the peripheral blood repopulates (Fig. 1E). Although no significant modulation of leukocyte number was observed within the bone marrow (Fig. 1C), a similar, yet delayed, trend followed the peripheral blood neutrophils with a decrease in cell numbers in the bone marrow approximately 24 h after that seen in the peripheral blood. This is likely to account for leukocyte mobilization to repopulate the peripheral circulation (Fig. 1F).

Analysis of peritoneal lavage fluid by ELISA revealed baseline levels of 2.155 ± 0.450 ng/ml Gal-3 within the cavity. These levels increased significantly during the peritonitis time course, with peak levels at 72 h (7.293 ± 1.239 ng/ml) (Fig. 1G). Endogenous expression of Gal-3 was minimal in monocyte/macrophages within the bone marrow and peripheral blood, but levels significantly increased in exudate peritoneal cells, with peak expression observed at 72 h (Fig. 1H). As expected, low levels of Gal-3 were detected in both peripheral blood [7, 9] and bone marrow–derived neutrophils; however, total levels were significantly increased in permeabilized, exudated neutrophils for 72 h, with peak expression observed at 24 h (Fig. 1I and J).

### Cellular localization of Gal-3 is modulated in neutrophils during inflammation

**Figure 2.**
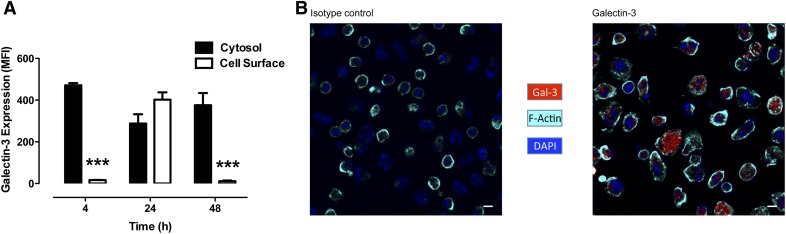
Cytosolic and cell surface expression of Gal-3 in murine neutrophils taken from the peritoneal exudate at 4, 24, and 48 h after zymosan ip. Surface Gal-3 expression was measured by flow cytometry on Ly6G^+^ neutrophils, and total Gal-3 expression was measured after permeabilization. (A) Cytosolic expression was calculated by subtracting the cell surface expression from the total expression. (B) Gal-3 expression in neutrophils taken from the peritoneal exudate at 4 h after zymosan as detected by confocal microscopy. MFI, mean fluorescence intensity. Scale bar = 5 µm. *N* = 8–12/group. ***P < 0.001 vs. cytosol.

To determine the cellular localization of Gal-3, flow cytometry was performed on nonpermeabilized vs. permeabilized neutrophils (**Fig. 2A**). At 4 h after zymosan, the Gal-3 expressed by murine neutrophils was predominantly intracellular; however, at 24 h, the protein was readily detected on the cell surface, and by 48 h, Gal-3 was again expressed predominantly in the intracellular compartment of the cell. Confocal analysis of the peritoneal neutrophils taken at the 4-h time point confirmed the intracellular localization of Gal-3 within the migrated neutrophils (Fig. 2B).

**Figure 3.**
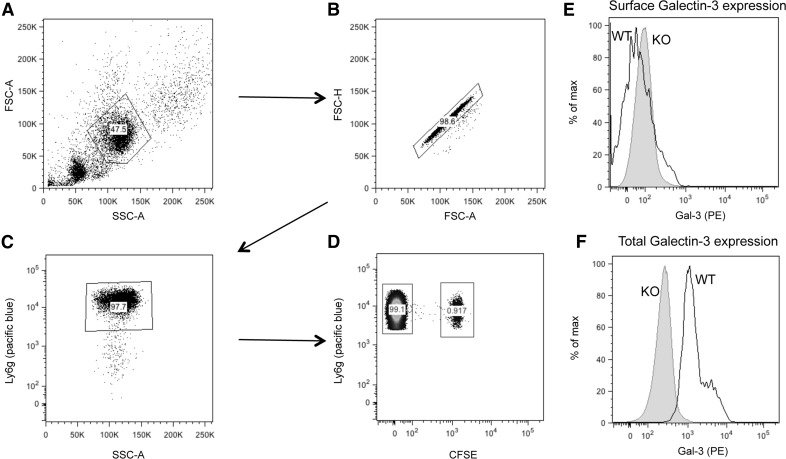
WT neutrophils exhibit increased Gal-3 expression upon transmigration to the peritoneal cavities of Gal-3–null mice. Bone marrow neutrophils were isolated from WT mice, labeled with CFSE, and administered i.v. to Gal-3–null mice, which subsequently underwent a zymosan (1 mg)–induced peritonitis. Peritoneal cavities were lavaged at 4 h and analyzed by flow cytometry. (A) Neutrophils were identifiable by their characteristic forward/side light scatter (FSC/SSC) profile. Following doublet exclusion (B), Ly6G^+^ neutrophils were identified (C). (D) WT Ly6G^+^ neutrophils were identified by their positive CFSE staining. Surface (E) and total (F) Gal-3 expression was measured on CFSE^−^ (neutrophils from Gal-3–null mice) and CFSE^+^ cells (neutrophils from WT mice) by flow cytometry, and representative histograms are shown. Max, maximum; PE, peritoneal exudate.

To determine whether the intracellular Gal-3 detected was derived from the extracellular environment, that is, being taken up from the inflammatory exudate or being produced by the neutrophils themselves, an adoptive transfer experiment was performed. CFSE-labeled bone marrow neutrophils isolated from WT mice were injected i.v. into Gal-3–null mice, and a zymosan-induced peritonitis performed. Neutrophils within lavage fluid were identified based on their characteristic forward/side light scatter and following doublet exclusion, Ly6G+ neutrophils were identified (**Fig. 3A-C**). CFSE-labeled Ly6G^+^ neutrophils were clearly detectable within peritoneal lavages, as shown in Fig. 3D. Negligible levels of Gal-3 were detectable on the neutrophil surface (Fig. 3E), whereas Gal-3 was readily detectable in permeabilized cells (Fig. 3F), indicating the presence of intracellular Gal-3 as shown in Fig. 1J.

### The role of Gal-3 in neutrophil clearance

**Figure 4.**
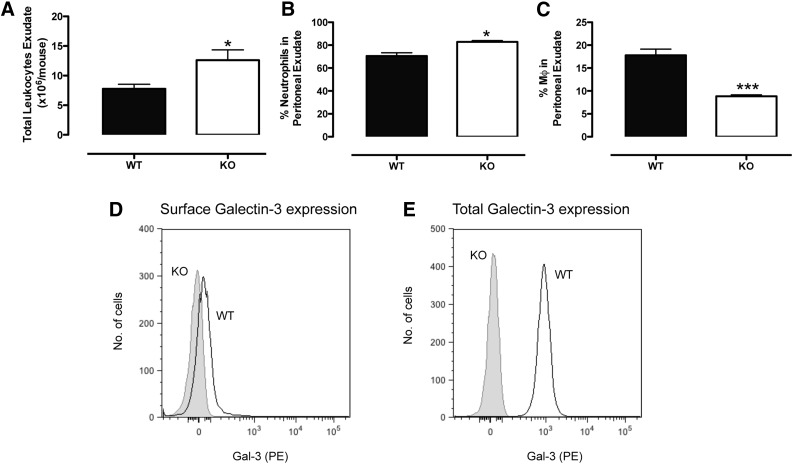
Gal-3–null mice demonstrate altered leukocyte trafficking. Total leukocyte counts (A), percentage of neutrophils (B), and percentage of macrophages (C) in the peritoneal exudate at 4 h after zymosan (1 mg) challenge. Surface (D) and total (E) Gal-3 expression was measured on Ly6G^+^ neutrophils by flow cytometry and representative histograms are shown. *N* = 4–7/group; *P < 0.05, ***P < 0.0001.>

To further investigate the role of neutrophil-derived Gal-3, the zymosan-induced peritonitis was repeated in Gal-3–null mice, initially at the 4 h point. That time point was chosen because it represents the peak of neutrophil infiltration into the cavity, which corresponds with low monocyte/macrophage numbers. Surprisingly, significantly higher numbers of leukocytes were observed in the cavities of Gal-3–null mice (**Fig. 4A**). Upon further examination, the percentage of neutrophils within the cavity was significantly higher in KO mice (Fig. 4B), whereas the percentage of macrophages was significantly lower (Fig. 4C). As shown in Fig. 2, surface expression of Gal-3 by peritoneal neutrophils was negligible (Fig. 4D), whereas it was readily detected in permeabilized cells by flow cytometry (Fig. 4E).

**Figure 5.**
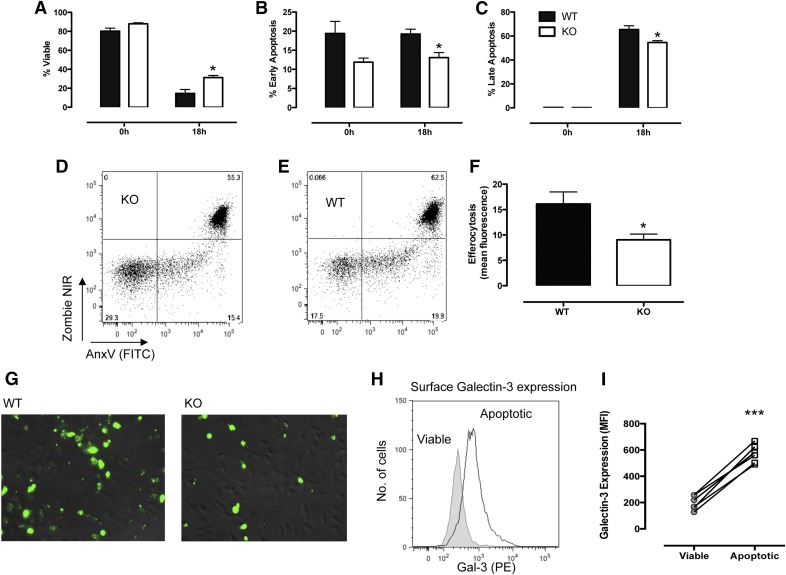
Gal-3–null mice demonstrate reduced levels of apoptosis. Percentage of viable cells (A), early apoptotic cells (B), and late apoptotic cells (C) after overnight culture of peritoneal exudate cells. Representative dot-plots after overnight culture of peritoneal exudate cells (taken 4 h after zymosan) showing cell viability (Zombie NIR) and phosphatidylserine exposure (Annexin V [AnxV]) in KO (D) and WT (E) mice exudated leukocytes. WT biogel-elicited macrophages were incubated with BODIPY-FL–labeled peritoneal exudate cells after overnight culture. (F) Efferocytosis was quantified using ImageJ software. (G) Representative images used for the data analysis are shown. Gal-3 levels were assessed on the surface of viable (AnxV^−^/Zombie^−^) and late apoptotic (AnxV^+^/zombie^+^) cells by flow cytometry (H and I). *N* = 4–7/group; *P < 0.05, ***P < 0.0001.

Neutrophil recruitment in Gal-3–null mice appears to vary in its magnitude depending on the inciting stimuli and the site of the inflammation. We, therefore, performed a peritonitis experiment in which we used *E. coli* instead of zymosan. The magnitude of the response was lower overall in neutrophil recruitment, and there were no differences between the 2 genotypes of mice in numbers of neutrophils recruited to the peritoneal cavity (data not shown). Again, Gal-3 was readily detectable within the cytosol of neutrophils that had traveled to the peritoneum in response to both *E. coli* and zymosan (Supplementary Fig. 1), whereas levels were low on the surface of zymosan-elicited neutrophils as well as on those elicited by *E. coli*. To investigate whether neutrophils lacking Gal-3 undergo apoptosis at a different rate than cells from WT animals, neutrophils collected from the peritoneal cavity were cultured overnight, and levels of apoptosis were assessed by flow cytometry. Upon removal from the cavity, most neutrophils were viable in both genotypes (**Fig. 5A**). However, after overnight culture, significantly fewer neutrophils from the Gal-3–null mice had undergone apoptosis (Fig. 5B–E). Because Gal-3 has been implicated in the clearance of neutrophils, experiments were conducted to investigate the role of neutrophil-derived Gal-3 on efferocytosis, and significantly reduced efferocytosis of Gal-3–null neutrophils was observed compared with WT neutrophils (Fig. 5F and G). Interestingly surface expression of Gal-3 was found to be increased on apoptotic neutrophils (Annexin V/Zombie double-positive cells), when compared with viable cells (Annexin V/Zombie double-negative cells) (Fig. 5 H and I).

### Role of Gal-3 during the resolving phase of the peritonitis model

**Figure 6.**
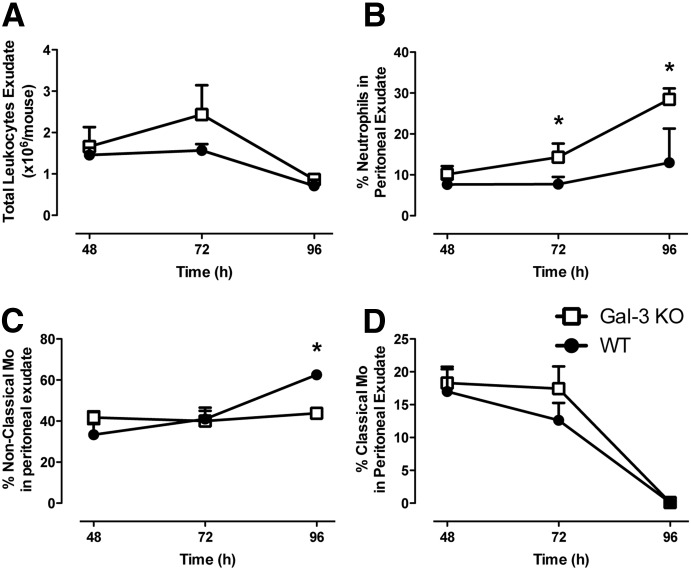
Gal-3–null mice demonstrate dysregulated resolution of inflammation compared with WT controls. Gal-3–null mice and age- and sex-matched WT controls were injected with 1 mg zymosan i.p., and peritoneal exudates were collected during the resolution period (48–96 h). Total leukocyte counts (A), percentage of neutrophils (B), percentage of nonclassic monocytes (Mo) (C), and percentage of classic monocytes (D) in peritoneal exudates after zymosan (1 mg) administration. *N* = 5/group; *P < 0.05.

Because there is evidence in the literature that Gal-3 is involved in neutrophil clearance, a key facet of the resolution process, leukocyte trafficking was compared in Gal-3–null mice and their WT counterparts at 48–96 h after zymosan. At 48 h after zymosan, total leukocyte counts were comparable for WT and Gal-3–null mice; however, at 72 h, there was a trend toward more leukocytes in the peritoneal cavities of Gal-3–null mice, although that increase did not reach statistical significance, and levels had declined to those observed in WT mice by 96 h (**Fig. 6A**). Interestingly, the percentage of exudated neutrophils was significantly higher in Gal-3–null mice at the 72 and 96 h points (Fig. 6B), indicating a potential defect in neutrophil clearance in these mice. Assessment of apoptosis by Annexin V/propidium iodide staining revealed no differences between the 2 genotypes of mice in the levels of apoptotic cells at these later times (**Table 1**). Because monocytes, particularly nonclassic monocytes, are important in the resolution of inflammation, the numbers of both subsets were compared between the strains of mice. Approximately 40% of cells recovered were nonclassic monocytes in both genotypes at 48 h, and that 40% remained stable in Gal-3–null mice up to 96 h. This was in contrast to WT mice in which the number of nonclassic monocytes significantly increased at 96 h (Fig. 6C). In contrast, 20% of the cells recovered from the peritoneum were classic monocytes in both genotypes, and that was not significantly altered at 72 h, although a trend toward fewer classic monocytes was seen in the WT mice, with approximately 15% of total leukocytes being classic monocytes. By 96 h, the number of classic monocytes was negligible in both genotypes of mice (Fig. 6D).

**TABLE 1.** Levels of apoptosis in neutrophils recruited to the peritoneal cavity of mice after zymosan administration

Time (h)	Early apoptotic (AnxV^+^/PI^−^)		Late apoptotic (AnxV^+^/PI^+^)	
	WT	Gal-3–null	WT	Gal-3–null
48	15.9 ± 1.5	18.8 ± 2.0	71.1 ± 3.2	67.9 ± 2.2
72	15.6 ± 1.2	17.1 ± 1.7	73.8 ± 1.8	69.4 ± 2.6
96	3.6 ± 0.9	2.5 ± 0.4	8.6 ± 2.1	7.3 ± 1.8

Data are expressed as percentage of early or late apoptosis (means ± sem). AnxV, Annexin V; PI, propidium iodide. *N* = 3–4 mice/group.

## DISCUSSION

The modulation of the inflammatory process, through effects on immune cell biology, by members of the galectin family is an ever-growing area of research. The focus has, however, been largely on the T cell–driven models of autoimmune disease and, in the case of Gal-3, in cancer. There are several reports describing the effects of Gal-3 on neutrophil activation and its importance in models of infection, in which it might function as an alarmin by augmenting the inflammatory response [21]. With this study, we expanded current knowledge of the role of Gal-3 in acute inflammation, identifying a role for the endogenous protein in neutrophil apoptosis and clearance.

Previous studies have failed to detect expression of Gal-3 intracellularly in murine neutrophils, although it is readily detectable on the cell surface [7, 11]. This has led to the assumption that the effects on neutrophil recruitment in Gal-3–null mice are due to a lack of extracellular Gal-3, likely released from cells such as inflammatory macrophages. We have confirmed that bone marrow and murine neutrophils from naïve, peripheral blood express low levels of Gal-3. However, in contrast to previous findings, we have shown that upon migration to the inflamed peritoneal cavity, intracellular Gal-3 levels are significantly increased in neutrophils.

Struck by this significant increase in Gal-3 expression upon migration to the inflammatory site, we have sought to address the role of neutrophil Gal-3 through investigation of the profile of neutrophil recruitment, apoptosis, and clearance in the zymosan peritonitis model. Increased levels of neutrophil recruitment into the peritoneal cavity of Gal-3–null mice were observed at 4, 72, and 96 h. This is in contrast to the reported response to thioglycollate in previous studies, as well as in models of pneumonia, in which neutrophil migration to the peritoneal cavity and lungs, respectively, was reduced in Gal-3–null mice [6, 9, 11, 12]. Such discrepancies may be due to the inducing inflammatory stimuli because, similar to zymosan, *E. coli* infection also resulted in a significant enhancement of neutrophil trafficking to the lungs of Gal-3–null mice [9]. The finding that *E. coli* increases neutrophil migration to the lungs in Gal-3–null mice is in contrast to our findings in the peritoneal cavity. This highlights the differences among distinct anatomic sites and might be due to the effects of Gal-3 on stromal cells at different sites. A different strain of mice was used in this study, which may also account for the observed differences. Further similarities exist between these inciting stimuli, which may account for the differing responses seen. *Streptococcus pneumoniae* infection increases Gal-3 levels in bronchoalveolar lavage fluid as high as 50 μg/ml; likewise, cutaneous infection with *L. major* LV39 results in increased levels of extracellular Gal-3 and is associated with enhanced neutrophil recruitment. In contrast, infection with *E. coli* or the *L. major* substrain Friedlin failed to significantly induce Gal-3 release, and a role for Gal-3–dependent neutrophil recruitment was not observed. Although we observed Gal-3 within the peritoneal exudate and levels were significantly increased during the course of the response, the amount detectable was in the low nanograms per milliliter range, and similar to what was observed after *E. coli* infection, neutrophil recruitment was increased, rather than decreased, in Gal-3–null mice. Another important factor might be the mechanism by which neutrophils travel to the inflammatory site. Migration in response to zymosan and *E. coli* is known to be dependent on β2 integrins [22], which function to allow neutrophils to adhere to and crawl on the endothelium [23]. In contrast, neutrophil trafficking to the lungs in response to *S. pneumoniae* is independent of β2 integrins, and it is thought that, under these circumstances, Gal-3 is able to act as an adhesion molecule to facilitate neutrophil trafficking, which likely explains the requirement for high levels of the protein extracellularly in these models. Our data suggest that the observed enhancement in neutrophil numbers within the peritoneal cavity of Gal-3–null mice is due to alterations in neutrophil clearance, rather than a direct role on neutrophil trafficking per se, although a direct role cannot be ruled out.

It is important to consider the cellular localization of galectins when studying their actions. Published reports on the effects of Gal-3 have shown cellular localization affects its function in the induction of apoptosis in T cells. Extracellular Gal-3 induces T cell apoptosis [24], whereas intracellular Gal-3 inhibits apoptosis [25]. For neutrophils, extracellular Gal-3 delays spontaneous apoptosis, although no role has been identified for the intracellular protein in murine neutrophils with similar rates of apoptosis observed between WT and Gal-3–null cells [6]. This study demonstrated intracellular expression of Gal-3 at 4 h after zymosan administration, and importantly, our data show an increased surface expression of Gal-3 on apoptotic neutrophils. These findings, together with the delayed apoptosis observed in Gal-3–null neutrophils recovered from the peritoneal cavity, lead us to hypothesize that Gal-3 is externalized by neutrophils and acts as an “eat-me” signal. Because efferocytosis peaks in this model at 6 h after zymosan administration [26], it would be expected that more neutrophils would be retained in the peritoneal cavity of Gal-3–null mice at the 4 h time point. The role of Gal-3 as an “eat-me” signal also explains why there are fewer cells in the peritoneal cavity with surface Gal-3 at later times (48 h onward) because these cells will have been cleared from the peritoneal cavity. Our findings are in contrast to the aforementioned study by Farnworth et al. [6] and the study of Colnot et al. [11], in which no defect in apoptosis was observed in neutrophils from Gal-3–null mice. There are, however, differences among the studies; importantly, in our study, apoptosis was assessed after incubation of neutrophils that had emigrated to the inflamed peritoneal cavity, whereas bone marrow neutrophils were used in the study of Farnworth et al. [6]. Although Colnot et al. [11] assessed levels of apoptosis in neutrophils taken from the peritoneal cavity, this was in a model of thioglycollate-induced peritonitis, and cells were taken 24 h after administration, whereas we observed reduced levels of apoptosis in cells taken at 4 h. Similar to the findings of Colnot et al. [11], we did not observe differences in the levels of apoptosis in cells collected from the peritoneum at later times (48–96 h). During the course of the inflammatory response, we found that the cellular localization of Gal-3 was altered. As mentioned above, Gal-3 levels were increased in the cytosol of neutrophils present within the peritoneal cavity at the 4-h point. Our data from the adoptive transfer experiment indicate that the neutrophils themselves are a source of Gal-3, although that does not rule out the possibility that neutrophils can bind Gal-3 present within the extracellular environment. In fact Karlsson et al. [13] showed that apoptotic neutrophils readily bound recombinant Gal-3. At 24 h, Gal-3 was detectable on both the cell surface and intracellularly; we hypothesize that satiated neutrophils might translocate Gal-3 to the cell surface to signal to incoming phagocytes that they need to be cleared. This is supported by the observed reduction in efferocytosis of Gal-3–null neutrophils by WT biogel-elicited macrophages and the lack of neutrophils with surface Gal-3 detectable at 48 h after zymosan.

Macrophages are crucial to the resolution process, with essential roles in the clearance of apoptotic neutrophils [27]. Previous studies have demonstrated defective resolution processes in Gal-3–null mice, with reduced alternative monocyte activation and, consequently, reduced phagocytic capabilities of these cells [28, 29], which correlates with our data showing reduced numbers of nonclassic monocytes within the peritoneal cavity of Gal-3–null mice during the resolution phase.

Together, our data highlight a role for endogenous Gal-3 in the host phagocyte function, enhancing neutrophil apoptosis and clearance. Failure to eliminate dying neutrophils leads to tissue damage and dissemination of cellular contents, which can have major pathologic consequences, particularly in infection.

## AUTHORSHIP

R.D.W performed experiments, analyzed data, and wrote the manuscript. P.R.S., P.T., and M.B.F. performed experiments and analyzed data. L.V.N. performed experiments, analyzed data, and wrote the manuscript, and D.C. designed and performed experiments, analyzed data, and wrote the manuscript.

## Supplementary Material

Supplemental Data

## Abbreviations

Gal-3: galectin-3
KO: knockout
WT: wild-type
