# Supplementary material for: Galectin-3–null mice display defective neutrophil clearance during acute inflammation
Source: J Leukoc Biol. 2016 Oct 12;101(3):717–26. doi: 10.1189/jlb.3A0116-026RR (PMC5295850; doi:10.1189/jlb.3A0116-026RR)
Supplement: Supplemental Data [file supp_101_3_717__index.html]

Galectin-3–null mice display defective neutrophil clearance during acute inflammation — Galectin-3–null mice display defective neutrophil clearance during acute inflammation — Supplemental Data 

# Galectin-3–null mice display defective neutrophil clearance during acute inflammation

## Supplemental Data

- Supplemental Data
