# Supplementary figures and images for: Galectin-3–null mice display defective neutrophil clearance during acute inflammation
Source: J Leukoc Biol. 2016 Oct 12;101(3):717–26. doi: 10.1189/jlb.3A0116-026RR (PMC5295850; doi:10.1189/jlb.3A0116-026RR)

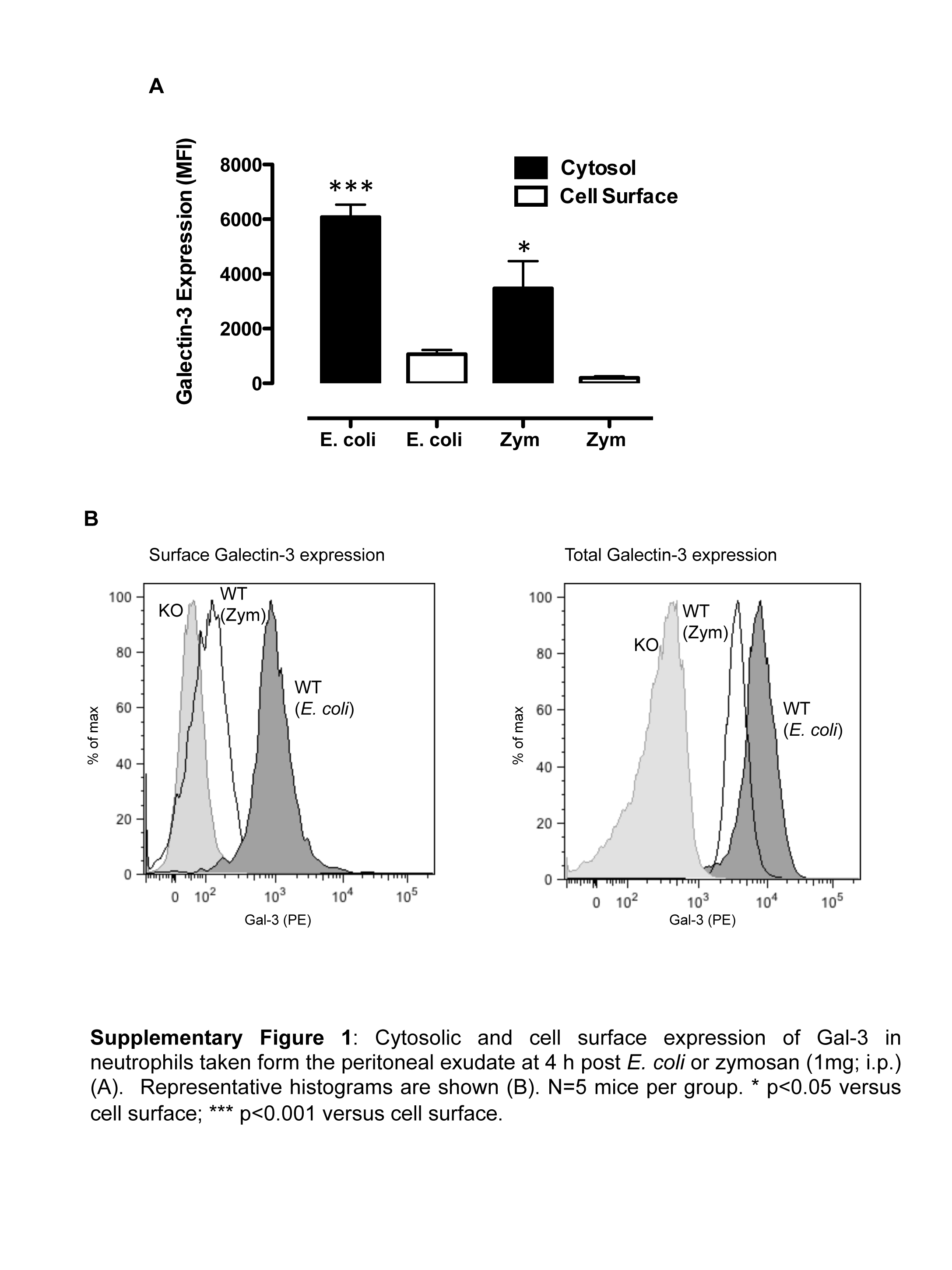

Supplement: Supplemental Data [file supp_jlb.3A0116-026RR_Supplemental_Figure.tif]
